# IL-33, IL-25 and TSLP contribute to development of fungal-associated protease-induced innate-type airway inflammation

**DOI:** 10.1038/s41598-018-36440-x

**Published:** 2018-12-21

**Authors:** Yoshihisa Hiraishi, Sachiko Yamaguchi, Takamichi Yoshizaki, Aya Nambu, Eri Shimura, Ayako Takamori, Seiko Narushima, Wakako Nakanishi, Yosuke Asada, Takafumi Numata, Maho Suzukawa, Yasuhiro Yamauchi, Akira Matsuda, Ken Arae, Hideaki Morita, Tomoaki Hoshino, Hajime Suto, Ko Okumura, Kenji Matsumoto, Hirohisa Saito, Katsuko Sudo, Motoyasu Iikura, Takahide Nagase, Susumu Nakae

**Affiliations:** 1 Laboratory of Systems Biology, Center for Experimental Medicine and Systems Biology, The Institute of Medical Science, The University of Tokyo, Tokyo, 108-8639 Japan; 2 Department of Respiratory Medicine, Graduate School of Medicine, The University of Tokyo, Tokyo, 113-8655 Japan; 3 Atopy Research Center, Juntendo University School of Medicine, Tokyo, 113-8412 Japan; 4 Department of Ophthalmology, Juntendo University School of Medicine, Tokyo, 113-8412 Japan; 5 Department of Cardiovascular Surgery, Saitama Medical Center, Jichi Medical University, Saitama, 330-8503 Japan; 6 National Hospital Organization Tokyo National Hospital, Tokyo, 204-8585 Japan; 7 Department of Allergy and Clinical Immunology, National Research Institute for Child Health and Development, Tokyo, 157-8535 Japan; 8 The 1st Department of Medicine, Kurume University School of Medicine, Fukuoka, 830-0011 Japan; 9 Animal Research Center, Tokyo Medical University, Tokyo, 160-8402 Japan; 10 Department of Respiratory Medicine, National Center for Global Health and Medicine, Tokyo, 162-8655 Japan; 11 Precursory Research for Embryonic Science and Technology (PRESTO), Japan Science and Technology Agency, Saitama, 332-0012 Japan

## Abstract

Certain proteases derived from house dust mites and plants are considered to trigger initiation of allergic airway inflammation by disrupting tight junctions between epithelial cells. It is known that inhalation of proteases such as house dust mite-derived Der p1 and/or papaya-derived papain caused airway eosinophilia in naïve mice and even in *Rag*-deficient mice that lack acquired immune cells such as T, B and NKT cells. In contrast, little is known regarding the possible involvement of proteases derived from *Aspergillus* species (fungal-associated proteases; FAP), which are ubiquitous saprophytic fungi in the environment, in the development of allergic airway eosinophilia. Here, we found that inhalation of FAP by naïve mice led to airway eosinophilia that was dependent on protease-activated receptor-2 (PAR2), but not TLR2 and TLR4. Those findings suggest that the protease activity of FAP, but not endotoxins in FAP, are important in the setting. In addition, development of that eosinophilia was mediated by innate immune cells (ILCs) such as innate lymphoid cells, but not by acquired immune cells such as T, B and NKT cells. Whereas IL-33, IL-25 and thymic stromal lymphopoietin (TSLP) are involved in induction of FAP-induced ILC-mediated airway eosinophilia, IL-33—rather than IL-25 and/or TSLP—was critical for the eosinophilia in our model. Our findings improve our understanding of the molecular mechanisms involved in induction of airway inflammation by FAP.

## Introduction

Atopic asthma is characterized by eosinophilic airway inflammation, mucus overproduction and bronchial hyperreactivity that lead to physiological and structural remodeling events in the lung^1,2^. Exposure to airborne allergens derived from animals, arthropods and fungi is considered to be an important risk factor for development of asthma^3–5^. Certain protease allergens, such as Der p1 derived from house dust mites (HDM), are considered to be triggers that activate epithelial cells via protease-activated receptors (PARs) and/or Toll-like receptors (TLRs)^6–8^, followed by activation of the innate immune system. In particular, it is known that an HDM-derived cysteine protease, Der p1, can disrupt tight junctions between epithelial cells as a result of its protease activity^2,9^. In addition, Der p1 can induce cell death (necrosis) of epithelial cells, which then release damage-associated molecular patterns (DAMPs; also called alarmins) such as HMGB-1, IL-1α, IL-33, uric acid and ATP^2,10^. Those alarmins immediately induce inflammation by activating immune cells. Inhalation of a plant-derived cysteine protease, papain, which is homologous to HDM-derived Der p1^11^, by humans resulted in development of asthma-like airway inflammation^12^. Likewise, inhalation of papain and/or Der p1 induced airway eosinophilia in naïve mice and even in *Rag*-deficient mice that lack acquired immune cells such as T, B and NKT cells^13,14^. In the setting, IL-33 derived from alveolar epithelial cells damaged by papain acts as a DAMP that activates group 2 innate lymphoid cells (ILC2) and basophils, followed by induction of type 2 cytokine-dependent eosinophilia^13–16^.

Spores and/or conidia from *Aspergillus* species, which are ubiquitous saprophytic fungi in the environment^17,18^, are thought to contribute to development of atopic asthma^19–21^. However, airway neutrophilia, rather than eosinophilia, was observed in mice at 18 hours after a single inhalation of proteases derived from *Aspergillus* species (fungal-associated proteases; FAP)^22^. We previously reported that inhalation of high–dose papain resulted in infiltration of neutrophils, whereas inhalation of the optimal dose of papain resulted in infiltration of eosinophils, into BAL fluids of naïve mice^16^. Therefore, we hypothesized that inhalation of FAP also may result in infiltration of eosinophils into BAL fluids of naïve mice. In fact, we found that over a certain concentration range FAP induced airway eosinophilia in mice, suggesting involvement of FAP in development of asthma^13,14^. Therefore, in the present study, we investigated the mechanisms underlying airway eosinophilia induced by FAP.

## Results

### Fungal-associated proteases (FAP) induce asthma-like airway eosinophilia in mice without prior sensitization

**Figure 1:**
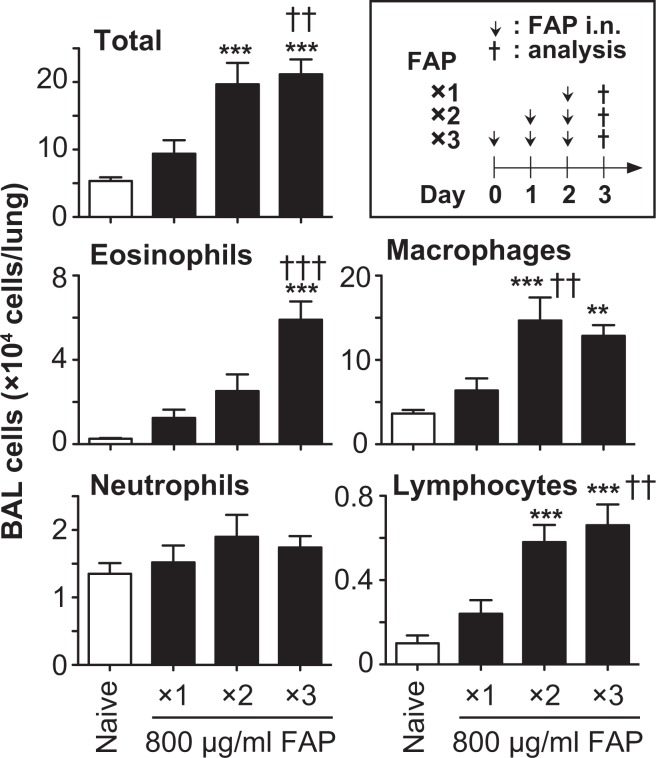
Induction of airway eosinophilia in naïve mice after FAP inhalation. Naïve C57BL/6-wild-type mice were treated intranasally with or without 800 μg/ml FAP once per day for 1 to 3 days (see the box). Twenty-four h after the last FAP inhalation, BAL fluids were collected. The numbers of various leukocytes in each BAL fluid were determined. Data show the mean ± SEM (naïve mice, n = 8; FAP-treated mice, n = 10), and representative results that were obtained in 2 independent experiments. **p < 0.005 and ***p < 0.0005 vs. the corresponding values for naive mice, and ^††^p < 0.005 and ^†††^p < 0.0005 vs. the corresponding values for FAP-treated mice (×1).

Others observed infiltration of neutrophils rather than eosinophils in BAL fluids of naïve mice at 18 hours after one inhalation of FAP^22,23^. We previously reported that inhalation of a high dose of papain resulted in infiltration of neutrophils, whereas inhalation of the optimal dose of papain resulted in infiltration of eosinophils, into BAL fluids of naïve mice^16^. Therefore, we hypothesized that inhalation of FAP also may lead to infiltration of eosinophils into BAL fluids of naïve mice. Based on those reports, we attempted to establish a murine model of FAP inhalation-induced airway eosinophilia. C57BL/6 wild-type mice were intranasally treated with 800 μg/ml FAP once per day for 1 to 3 days (see the box in Fig. 1). Twenty-four hours after the last FAP inhalation, the cell profiles in BAL fluids were investigated. As shown in Fig. 1a, the numbers of eosinophils, neutrophils, macrophages and lymphocytes were slightly, but not significantly, increased after a single inhalation of FAP. Macrophages and lymphocytes, but not eosinophils or neutrophils, were significantly increased after two inhalations of FAP, while macrophages, lymphocytes and eosinophils were significantly increased after three inhalations of FAP (Fig. 1). Therefore, our model for studying the mechanisms of FAP-induced airway eosinophilia used mice treated intranasally with FAP once per day for 3 days.

**Figure 2:**
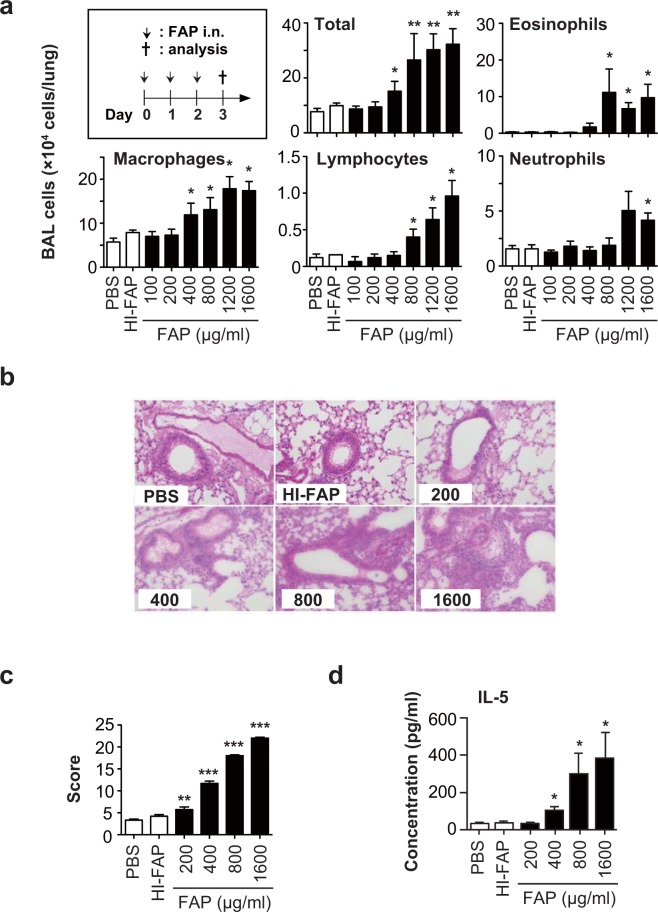
Importance of protease activity of FAP for induction of airway eosinophilia. Naïve C57BL/6-wild-type mice were treated intranasally with various concentrations of FAP (100–1600 μg/ml), heat-inactivated FAP (HI-FAP; 800 μg/ml) or PBS once per day for 3 days. Twenty-four h after the last inhalation, BAL fluids and lungs were collected. (**a**) Numbers of leukocytes in BAL fluids (PBS, n = 5; HI-FAP, n = 5; FAP, n = 4-5). (**b**) Lung sections stained with hematoxylin-eosin (×200). (**c**) Severity scores of lung inflammation (PBS, n = 6; HI-FAP, n = 9; FAP, n = 11–14). (**d**) The levels of IL-5 in BAL fluids by ELISA (PBS, n = 5; HI-FAP, n = 5; FAP, n = 5). Data show the mean ± SEM, and representative results that were obtained in 2 independent experiments. *p < 0.05, **p < 0.005 and ***p < 0.0005 vs. the corresponding values for PBS-treated mice.

Next, C57BL/6 wild-type mice were treated intranasally with various concentrations of FAP (100–1600 μg/ml), heat-inactivated FAP (100–1600 μg/ml) or PBS once per day for 3 days. In our pilot studies, we found that the numbers of leukocytes (total, macrophages, lymphocytes, neutrophils and eosinophils) in the BAL fluids of mice treated with 100, 200, 400, 800, 1200 and 1600 μg/ml heat-inactivated FAP were comparable to those of mice treated with PBS (data not shown). Therefore, we used 800 μg/ml heat-inactivated FAP in the later experiments. The numbers of various leukocytes in the BAL fluids and the severity of lung inflammation determined by histological analysis were not dramatically increased at the lowest concentrations of FAP (100 and 200 μg/ml) compared with PBS (Fig. 2a–c). Although the numbers of leukocytes in the BAL fluids were not significantly increased at 400 μg/ml FAP, lung inflammation accompanied by epithelial hyperplasia and mucus secretion was observed (Fig. 2a–c). Consistent with Fig. 1, a significant increase in eosinophils but not neutrophils was observed in BAL fluids from mice that inhaled 800 μg/ml FAP, while both eosinophils and neutrophils were significantly increased after inhalation of the highest FAP doses (1200 and 1600 μg/ml) (Fig. 2a). Asthma-like inflammation was seen in the lungs of mice treated with 800 μg/ml FAP, while chronic obstructive pulmonary disease-like inflammation associated with tissue destruction was observed at 1200 and 1600 μg/ml FAP (Fig. 2b,c; and data not shown). Similar observations were reported in mice treated with a cysteine protease, papain^16^. In that study, ELISA hardly detected cytokines in the BAL fluids of mice after papain inhalation because they had been cleaved by the protease activity of papain^16^. Likewise, using ELISA, we hardly detected IFN-γ, IL-4, IL-13 or IL-17A in the BAL fluids of mice after FAP inhalation (data not shown). On the other hand, we found that the levels of IL-5 were significantly increased in the BAL fluids of mice after inhalation of 800 μg/ml FAP (Fig. 2d).

**Figure 3:**
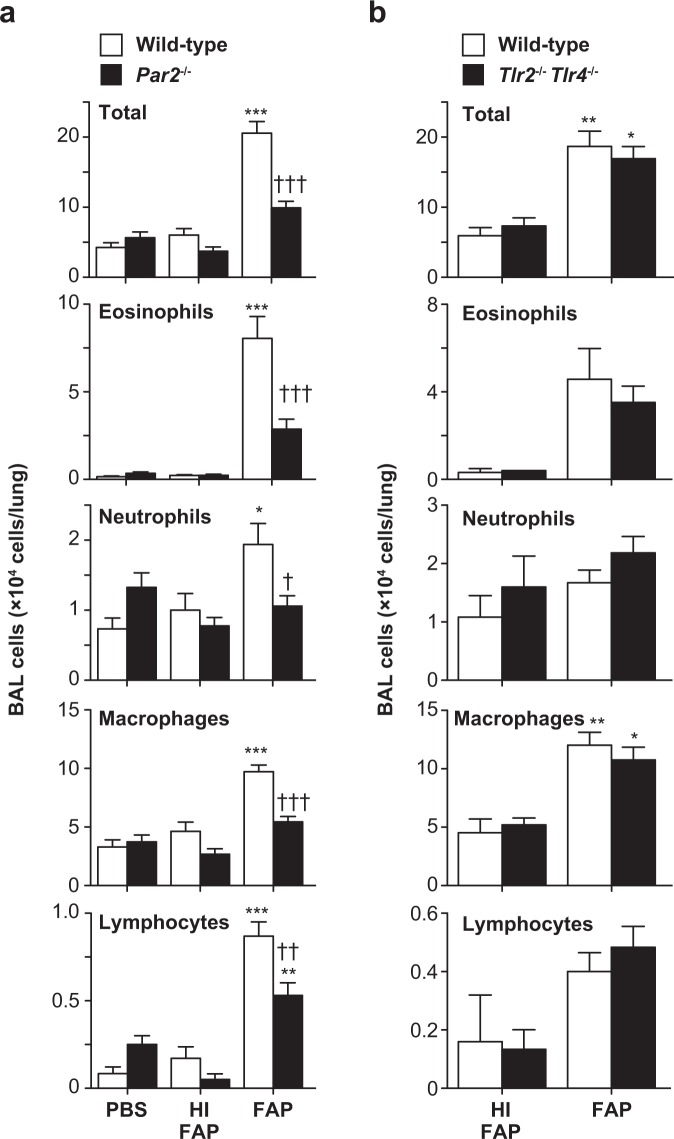
Importance of Par2, but not TLR2 or TLR4, for development of FAP-induced airway eosinophilia. Mice were treated intranasally with 800 μg/ml FAP, 800 μg/ml heat-inactivated FAP (HI-FAP) or PBS once per day for 3 days. Twenty-four h after the last inhalation, BAL fluids were collected. (**a**) Numbers of leukocytes in BAL fluids from C57BL/6-wild-type mice (PBS, n = 12; HI-FAP, n = 14; FAP, n = 32) and –*Par2*^−/−^ mice (PBS, n = 8; HI-FAP, n = 8; FAP, n = 20). (**b**) Numbers of leukocytes in BAL fluids from C57BL/6-wild-type mice (HI-FAP, n = 5; FAP, n = 17) and –*Tlr2*^−/−^*Tlr4*^−/−^ mice (HI-FAP, n = 3; FAP, n = 12). Data show the mean ± SEM, which are pooled from 2 independent experiments. *p < 0.05, **p < 0.005 and ***p < 0.0005 vs. the corresponding values for PBS-treated mice, and ^††^p < 0.005 and ^†††^p < 0.0005 vs. the corresponding values for FAP-treated wild-type mice.

Therefore, in our subsequent experiments, we used 800 μg/ml FAP to induce asthma-like airway eosinophilia in mice. In addition, inhalation of heat-inactivated FAP did not induce airway eosinophilia accompanied by eosinophilia or elevation of IL-5 in the BAL fluids (Fig. 2a–d). Those findings suggest that the protease activity of FAP is crucial for induction of asthma-like airway eosinophilia. In support of this, mice deficient in protease-activated receptor-2 (*Par2*^−/−^ mice) showed reduced inflammatory cell counts in the BAL fluids during FAP-induced airway eosinophilia (Fig. 3a). In addition, the number of BAL cells was comparable between wild-type and *Tlr2*^−/−^*Tlr4*^−/−^ mice during FAP-induced airway eosinophilia, suggesting that contamination by endotoxin does not influence the induction of FAP-induced airway eosinophilia (Fig. 3b).

### IL-33, but not IL-25 or TSLP, is critical for FAP-induced airway eosinophilia, independently of acquired immune cells

**Figure 4:**
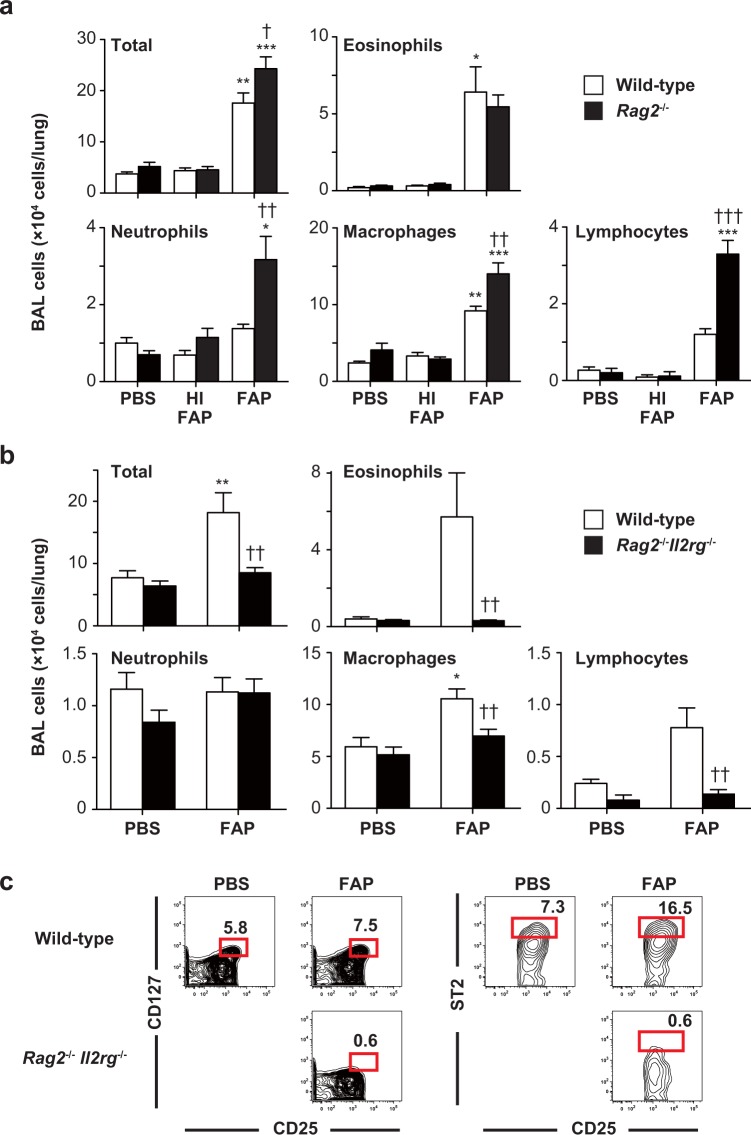
Importance of innate-type, but not acquired, immune cells for development of FAP-induced airway eosinophilia. Mice were treated intranasally with 800 μg/ml FAP, 800 μg/ml heat-inactivated FAP (HI-FAP) or PBS once per day for 3 days. Twenty-four h after the last inhalation, BAL fluids were collected. (**a**) Numbers of leukocytes in BAL fluids from C57BL/6-wild-type mice (PBS, n = 6; HI-FAP, n = 9; FAP, n = 20) and –*Rag2*^−/−^ mice (PBS, n = 5; HI-FAP, n = 8; FAP, n = 21). (**b**) Numbers of leukocytes in BAL fluids from C57BL/6-wild-type mice (PBS, n = 5; FAP, n = 9) and –*Rag2*^−/−^*Il2rg*^−/−^ mice (PBS, n = 5; FAP, n = 13). (**c**) Profiles of CD25^+^ CD127^+^ or CD25^+^ ST2^+^ ILC2s among 7-aminoactinomycin D-negative, lineage marker-negative CD45^+^ cells in lungs from C57BL/6-wild-type mice and –*Rag2*^−/−^*Il2rg*^−/−^ mice. Data show the mean + SEM, which are pooled from 2 independent experiments (**a**,**b**) and representative of similar results that were obtained in 3 independent experiments (**c**). *p < 0.05, **p < 0.005 and ***p < 0.0005 vs. the corresponding values for PBS-treated mice, and ^†^p < 0.05, ^††^p < 0.005 and ^†††^p < 0.0005 vs. the corresponding values for FAP-treated wild-type mice.

Infiltration of neutrophils into BAL fluids was seen even in *Rag1*^−/−^ mice at 18 hours after one inhalation of FAP, indicating that acquired immune cells such as T, B and NKT cells are not essential for that response^22^. In our model, as well, FAP-induced airway inflammation was observed in *Rag2*^−/−^ mice (Fig. 4a). In particular, neutrophils, macrophages and lymphocytes, but not eosinophils, were increased in BAL fluids of *Rag2*^−/−^ mice compared with wild-type mice (Fig. 4a), suggesting that acquired immune cells such as T, B and NKT cells were not essential for FAP-induced airway eosinophilia. On the other hand, FAP-induced airway eosinophilia was markedly impaired in *Rag2*^−/−^*Il2rg*^−/−^ mice, which lack acquired immune cells, innate lymphoid cells (ILCs) and IL-2, IL-4, IL-7, IL-9, IL-15 and IL-21, compared with wild-type mice (Fig. 4b), suggesting that ILCs and/or IL-2Rγ-ligands may be important for FAP-induced airway eosinophilia. We found that the number of group 2 ILCs (ILC2s) was increased in the lungs of wild-type mice, but not *Rag2*^−/−^*Il2rg*^−/−^ mice, after FAP inhalation (Fig. 4c).

**Figure 5:**
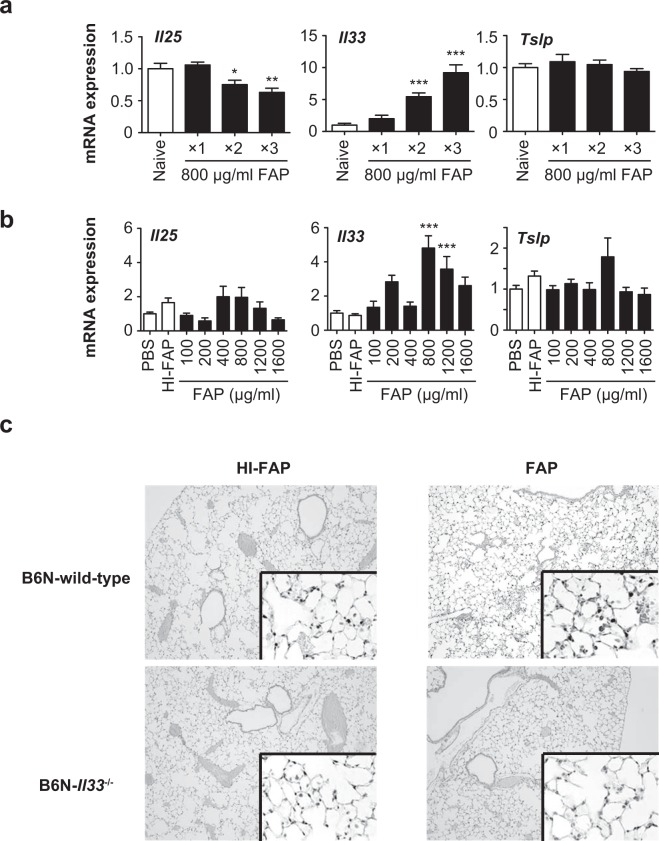
Increased expression of IL-33 in the lungs of FAP-treated mice. (**a**) Naïve C57BL/6-wild-type mice were treated intranasally with or without 800 μg/ml FAP once per day for 1 to 3 days as in Fig. 1. Twenty-four h after the last FAP inhalation, lungs were collected. mRNA expression levels of *Il25*, *Il33* and *Tslp* in the lungs were determined by quantitative PCR (naïve, n = 10; FAP, n = 10). (**b**) Naïve C57BL/6-wild-type mice were treated intranasally with various concentrations of FAP (100–1600 μg/ml), heat-inactivated FAP (HI-FAP; 800 μg/ml) or PBS once per day for 3 days as in Fig. 2a. Twenty-four h after the last inhalation, lungs were collected. mRNA expression levels of *Il25*, *Il33* and *Tslp* in the lungs were determined by quantitative PCR (PBS, n = 9–12; HI-FAP, n = 6–11; FAP, n = 6–10). (**c**) Mice were treated intranasally with 800 μg/ml FAP and heat-inactivated FAP (HI-FAP) once per day for 3 days. Twenty-four h after the last inhalation, lungs were collected. IL-33 expression in sections of the lungs was detected by immunohistochemistry. Blue = Mayer’s hematoxylin, and brown = anti-IL-33 Ab staining, respectively. ×10 and ×160 (inserted photos). Data show the mean ± SEM and representative results that were obtained in 2 independent experiments (**a**,**b**). *p < 0.05, **p < 0.005 and ***p < 0.0005 vs. the corresponding values for PBS-treated mice.

**Figure 6:**
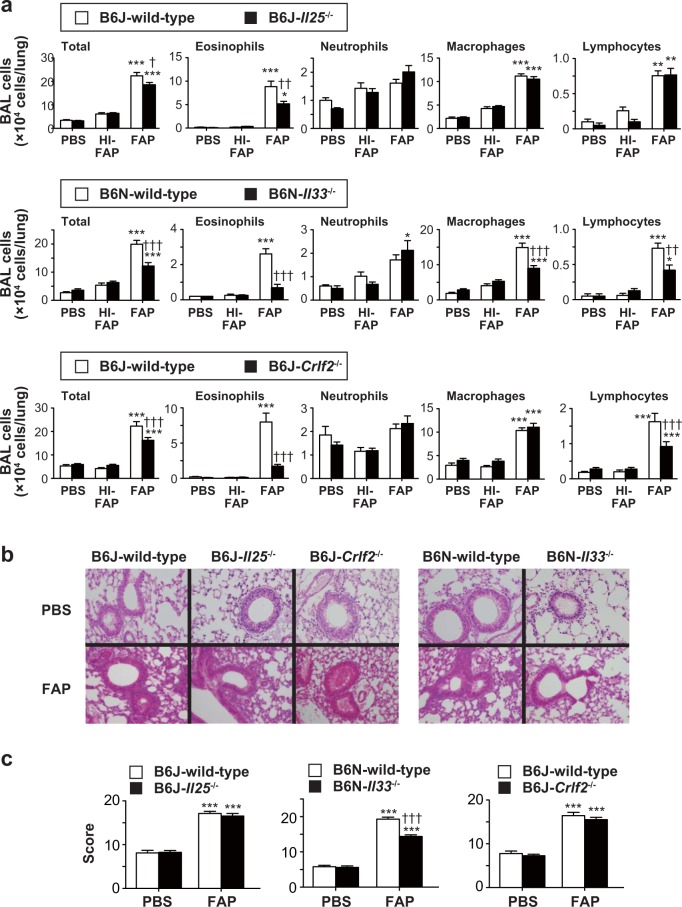
Involvement of IL-25, IL-33 and TSLP in development of FAP-induced airway eosinophilia. Mice were treated intranasally with 800 μg/ml FAP, 800 μg/ml heat-inactivated FAP (HI-FAP) or PBS once per day for 3 days. Twenty-four h after the last inhalation, BAL fluids and lungs were collected. (**a**) Numbers of leukocytes in BAL fluids from C57BL/6-wild-type mice (PBS, n = 8–12; HI-FAP, n = 10–14; FAP, n = 29–43), –*Il25*^−/−^ mice (PBS, n = 8; HI-FAP, n = 10; FAP, n = 52), –*Il33*^−/−^ mice (PBS, n = 8; HI-FAP, n = 11; FAP, n = 31) and –*Crlf2*^−/−^ mice (PBS, n = 10; HI-FAP, n = 13; FAP, n = 33). (**b**) Lung sections stained with hematoxylin-eosin (×200). (**c**)Severity scores of lung inflammation in C57BL/6-wild-type mice (PBS, n = 9–10; FAP, n = 11–19), –*Il25*^−/−^ mice (PBS, n = 8; FAP, n = 13), –*Il33*^−/−^ mice (PBS, n = 11; FAP; n = 11) and –*Crlf2*^−/−^ mice (PBS, n = 8; FAP, n = 10) Data show the mean + SEM, which are pooled from 2 or 3 independent experiments (**a**,**c**). *p < 0.05 and **p < 0.005 and ***p < 0.0005, the corresponding values for PBS-treated mice, and ^†^p < 0.05, ^††^p < 0.005 and ^†††^p < 0.0005 vs. the corresponding values for FAP-treated wild-type mice.

IL-25, IL-33 and TSLP are known to be produced by epithelial cells in the lungs^2,24^ and involved in activation of ILC2s to produce type 2 cytokines, thereby contributing to induction of type 2 cytokine-dependent inflammation such as airway eosinophilia^25,26^. We found that the mRNA expression level of *Il33*, but not *Il25* or *Tslp*, was significantly increased in the lungs of wild-type mice treated intranasally with 800 μg/ml FAP once per day for 1 to 3 days as shown in Fig. 1 (Fig. 5a), or with 0-1,200 μg/ml FAP once per day for 3 days as shown in Fig. 2 (Fig. 5b), in comparison with wild-type mice treated intranasally with PBS or heat-inactivated FAP. In association with this, the IL-33 protein levels were increased in the nuclei of alveolar epithelial cells from FAP-treated wild-type mice compared with heat-inactivated FAP-treated wild-type mice (Fig. 5c). To elucidate the contributions of IL-25, IL-33 and TSLP to induction of FAP-induced airway inflammation, *Il25*^−/−^, *Il33*^−/−^ and TSLP receptor-deficient (*Crlf2*^−/−^) mice were treated intranasally with 800 μg/ml FAP, 800 μg/ml heat-inactivated FAP or PBS once per day for 3 days. As shown in Fig. 6a, eosinophils were significantly decreased in the BAL fluids of all mutant mice (*Il25*^−/−^, *Il33*^−/−^ and *Crlf2*^−/−^ mice) compared with wild-type mice after the last FAP inhalation, while the neutrophil counts in the BAL fluids were comparable in all groups. In comparison with FAP-treated wild-type mice, macrophages were significantly reduced in the BAL fluid of FAP-treated *Il33*^−/−^, but not *Il25*^−/−^ or *Crlf2*^−/−^, mice (Fig. 6a). On the other hand, lymphocytes were significantly reduced in the BAL fluids of FAP-treated *Il33*^−/−^ and *Crlf2*^−/−^, but not *Il25*^−/−^, mice (Fig. 6a). In addition, the histological score of lung inflammation was significantly lower in FAP-treated *Il33*^−/−^, but not *Il25*^−/−^ or *Crlf2*^−/−^, mice than in FAP-treated wild-type mice (Fig. 6b,c). These observations suggest that IL-25, IL-33 and TSLP are crucial for FAP-induced airway eosinophilia, but IL-33 is more important than IL-25 and TSLP for FAP-induced airway inflammation accompanied by infiltration of macrophages and lymphocytes as well as eosinophils.

**Figure 7:**
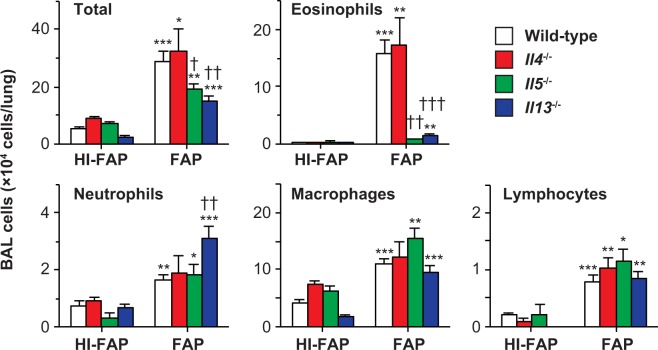
Importance of IL-5 and IL-13, but not IL-4, for development of FAP-induced airway eosinophilia. Mice were treated intranasally with 800 μg/ml FAP or heat-inactivated FAP (HI-FAP) once per day for 3 days. Twenty-four h after the last inhalation, BAL fluids were collected. Numbers of leukocytes in BAL fluids from C57BL/6-wild-type mice (HI-FAP, n = 9; FAP, n = 18), –*Il4*^−/−^ mice (HI-FAP, n = 5; FAP, n = 8), –*Il5*^−/−^ mice (HI-FAP, n = 3; FAP, n = 5) and –*Il13*^−/−^ mice (HI-FAP, n = 4; FAP, n = 10). Data show the mean + SEM. *p < 0.05, **p < 0.005 and ***p < 0.0005 vs. the corresponding values for HI-FAP-treated mice, and ^†^p < 0.05, ^††^p < 0.005 and ^†††^p < 0.0005 vs. the corresponding values for FAP-treated wild-type mice.

Type 2 cytokines such as IL-5 and IL-13 were shown to be important for IL-33-induced airway eosinophilia^13,27,28^. Therefore, we investigated involvement of type 2 cytokines in airway eosinophilia after FAP inhalation. The numbers of eosinophils in the BAL fluids were similar in *Il4*^−/−^ mice compared with wild-type mice after FAP inhalation (Fig. 7). On the other hand, eosinophils in the BAL fluids were greatly reduced in both *Il5*^−/−^ mice and *Il13*^−/−^ mice compared with wild-type mice (Fig. 7). These observations indicate that IL-5 and IL-13 are crucial for FAP-induced airway eosinophilia.

## Discussion

Protease allergens derived from HDM and/or fungi are considered to be important risk factors for development of allergic asthma. An HDM-derived cysteine protease, Der p1, can induce necrosis of epithelial cells, leading to release of DAMPs such as HMGB-1, IL-1α, IL-33, uric acid and ATP and then induction of local inflammation via activation of innate immune cells^2,10^. Inhalation of a plant-derived cysteine protease, papain, which is homologous to HDM-derived Der p1^11^, led to development of asthma-like airway inflammation^12^ in naïve mice and even *Rag*-deficient mice^13^. Likewise, airway inflammation was observed in naïve and *Rag*-deficient mice at 18 hours after a single inhalation of FAP derived from *Aspergillus* species^22^. In the setting, both neutrophils and eosinophils were recruited in the lungs, with neutrophils being dominant^22^. In addition, IL-17F, but not IL-17A, was shown to be important for induction of FAP-mediated airway neutrophilia^23^. On the other hand, it is unclear which cytokines are involved in FAP-induced airway eosinophilia, although STAT6, which is essential for IL-4 and IL-13 signals, and C3 were not required. To clarify this issue, we established a model of airway eosinophilia, rather than neutrophilia, by treating mice intranasally with a certain concentration range of FAP once per day for 3 days.

IL-25, IL-33 and TSLP, which are produced mainly by epithelial cells in airways, induce type 2 cytokines by various types of cells such as Th2 cells, NKT cells and/or ILC2^2,24–26^. Epithelial cell-derived IL-33 was reported to be crucial for papain-induced airway eosinophilia by production of type 2 cytokines by ILC2s and basophils^13–16^. Likewise, we found that epithelial cell-derived IL-33 was important for development of FAP-induced airway eosinophilia. Similar to *Il33*^−/−^ mice, *Il25*^−/−^ mice and *Crlf2*^−/−^ mice showed attenuated airway eosinophilia after FAP inhalation, suggesting that IL-25 and TSLP, in addition to IL-33, are involved in FAP-induced airway eosinophilia. On the other hand, after FAP inhalation the degree of inflammation in the lungs was similar in *Il25*^−/−^ mice and *Crlf2*^−/−^ mice, but significantly decreased in *Il33*^−/−^ mice, in comparison with wild-type mice. In addition, the expression levels of *Il33*, but not *Il25* or *Tslp*, mRNA were increased in the lungs of mice after FAP inhalation. IL-33 protein was also increased in the nuclei of alveolar epithelial cells in the setting. Since FAP-induced airway eosinophilia in *Par2*^−/−^ mice was attenuated, but not completely abrogated, induction of IL-33 in alveolar epithelial cells is both Par2-dependent and -independent. We previously demonstrated that IL-25 is produced by epithelial cells in the lungs and noses of mice during OVA-induced airway inflammation and HDM-induced allergic rhinitis, respectively^29,30^. On the other hand, in the lungs of FAP-treated mice, both IL-25 and TSLP were below the limit of immunohistochemical detection. These observations suggest that, rather than IL-25 and TSLP, IL-33 is the most important cytokine for development of FAP-induced airway inflammation.

Papain-induced, IL-33-mediated airway eosinophilia was reportedly mediated by ILC2s and basophils, but not by acquired immune cells such as T, B and NKT cells^13–16^. Similarly, we showed here that FAP-induced airway eosinophilia was markedly abrogated in *Rag2*^−/−^*Il2rg*^−/−^ mice, but developed normally in *Rag2*^−/−^ mice. These observations suggest that ILCs, but not T, B or NKT cells, may contribute in the setting, although we generated no direct evidence that IL-33-stimulated ILC2s induced airway eosinophilia in mice after FAP inhalation.

Taken together, we found that inhalation of FAP derived from *Aspergillus* resulted in induction of IL-33 in alveolar epithelial cells via the PAR-2 signaling pathway, but not the TLR2 or TLR4 pathway, followed by development of airway eosinophilia mediated by ILCs, but not T, B or NKT cells.

## Methods

### Mice

Wild-type mice (C57BL/6 J and C57BL/6 N, 7–11 weeks of age) were purchased from Japan SLC, Inc. (Shizuoka, Japan). C57BL/6J*-Par2*^−/−^ mice, -*Il4*^−/−^ mice and -*Il5*^−/−^ mice were obtained from Jackson Laboratory (Bar Harbor, MA, USA). C57BL/6*-Rag2*^−/−^ and *-Rag2*^−/−^*Il2rg*^−/−^ mice, C57BL/6*-Tlr2*^−/−^*Tlr4*^−/−^ mice and C57BL/6*-Il13*^−/−^ mice were kindly provided by Drs Hiromitsu Nakauchi (The University of Tokyo, Tokyo, Japan), Tsuneyasu Kaisho (RIKEN, Yokohama, Japan) and Andrew N.J. McKenzie (MRC, UK), respectively. C57BL6J-*Il25*^−/−^ mice, C57BL/6N-*Il33*^−/−^ mice and C57BL/6J-*Crlf2*^−/−^ mice were generated as described elsewhere^13,31,32^. All mice were maintained in a specific-pathogen-free environment at the Institute of Medical Science, The University of Tokyo. All animal experiments were approved by the Institutional Review Board of the Institute (A11-28 and A14-10) and conducted according to the ethical safety guidelines of the institution.

### Fungal-associated protease (FAP)-induced airway inflammation

Mice were placed under general anesthesia with isoflurane and then treated intranasally with 20 µl of 100–1600 μg/ml protease from *Aspergillus oryzae* (FAP; Sigma-Aldrich, St. Louis, MO, USA), 800 μg/ml heat-inactivated FAP (HI-FAP) that was prepared by incubation of FAP solution at 95 °C for 15 min in a block incubator, or PBS once per day for 1–3 days. The protease activities of 100–1600 μg/ml FAP, but not 100–1600 μg/ml HI-FAP, were observed by using Protease Activity Kit (Amplite^TM^ Universal Fluorimetric Protease Activity Kit; AAT Bioquest, Inc) (data not shown).

### Bronchoalveolar lavage fluids

Twenty-four h after the last challenge with FAP, HI-FAP or PBS, bronchoalveolar lavage (BAL) fluids were collected as described elsewhere^33,34^. The BAL fluids were centrifuged, and the BAL cells were resuspended in 200 μl of HBSS supplemented with 2% FCS. The number of each cell type among the BAL cells was counted with an automated hematology analyzer (XT-1800i; Sysmex, Hyogo, Japan), according to the manufacturer’s instructions.

### Quantitative PCR

Total RNA was extracted from lungs of mice 24 h after the last inhalation of FAP, HI-FAP or PBS, and cDNA was prepared as described elsewhere^34,35^. The expression levels of mRNA for cytokines in each sample were determined by the comparative Ct method after normalization with GAPDH expression using a CFX384 Touch qPCR System (Bio-Rad, Hercules, CA), as described elsewhere^34,35^. Primers, which were designed as shown in Table 1, were prepared by Eurofins Operon (Tokyo, Japan).

**Table 1:** Sequences of primers.

Gene	Forward (5′ → 3′)	Reverse (5′ → 3′)
*Il25*	GGCATTTCTACTCAGGAACGGA	GGTGGAGAAAGTGCCTGTGC
*Il33*	CAGGCCTTCTTCGTCCTTCAC	TCTCCTCCACTAGAGCCAGCTG
*Tslp*	CAATCCTATCCCTGGCTGCC	TGTGCCATTTCCTGAGTACCGT
*Gapdh*	CCCACTCTTCCACCTTCGATG	AGGTCCACCACCCTGTTGCT

### Histology

Twenty-four h after the last FAP inhalation, lungs were harvested and fixed in Carnoy’s solution. The fixed tissues were embedded in paraffin, sliced into 4-µm sections, and subjected to hematoxylin and eosin staining or periodic acid-Schiff staining. The severity of inflammation in lungs was scored as described elsewhere^34^.

### Immunohistochemistry

Twenty-four h after the last FAP inhalation, the trachea was cannulated with a 22-G needle attached to a syringe, and 4% paraformaldehyde was injected. Then the lungs were harvested and fixed in 4% paraformaldehyde at 4 °C overnight. The fixed tissues were embedded in paraffin and sliced into 4-µm sections. The sections were incubated with Blocking One Histo (0634904; Nacalai, Japan). After blocking, they were incubated with goat anti-mouse IL-33 polyclonal Ab (AF3626; Abcam plc, UK) in a humidified staining box at 4 °C overnight. They were then incubated with 0.3% hydrogen peroxide solution and 0.1% sodium azide for 20 minutes at room temperature to block endogenous peroxidase activities. Then the sections were incubated with horseradish peroxidase-conjugated secondary Ab (N-Histofine Simple Stain MAX PO Goat; 414351; Nichirei Biosciences Inc., Japan) for 30 minutes at room temperature. After washing, IL-33-producing cells in the lung sections were detected with an HRP/diaminobenzidine (DAB) system. Nuclei were counterstained with Mayer’s hematoxylin for 1 minute. The sections were mounted using Malinol (20092; Muto Pure Chemicals, Japan) and imaged using a BZ-X710 (KEYENCE, Japan).

### Flow cytometry

Lungs were minced and digested with Liberase-TM (Roche, Tokyo, Japan) and DNase (Roche, Tokyo, Japan) for 1 h at 37 °C. After filtration through a nylon mesh (40 μm cell strainer; Greiner, Tokyo, Japan), single lung cells were collected. The cells were incubated with anti-mouse CD16/CD32 mAb (93; eBioscience, San Diego, CA) for FcR blocking for 15 min on ice, and then incubated with each of the following: PE-conjugated anti-mouse CD3ε mAb (145-2C11, BioLegend, San Diego, CA), PE-conjugated anti-mouse Gr-1 mAb (RB6-8C5; eBioscience), PE-conjugated anti-mouse CD19 m Ab (6D; BioLegend), PE-conjugated anti-mouse NK1.1 mAb (PK136; BD Biosciences, San Jose, CA), PE-conjugated anti-mouse CD11b mAb (M1/70; BioLegend), PE-conjugated anti-mouse Ter-119 mAb (TER-119; BioLegend), PE/Cy7-conjugated anti-mouse CD25 mAb (PC61; BioLegend), Brilliant Violet 421-conjugated anti-mouse CD127 mAb (A7R34; BioLegend), Brilliant Violet 510-conjugated anti-mouse CD45 mAb (30-F11; BioLegend), and FITC-conjugated anti-Mouse ST2 mAb (DJ8, MD Bioscience, Zurich, Switzerland). After washing, the cells were suspended in HBSS supplemented with 2% FCS and 7-aminoactinomycin D. The proportions of CD127^+^ CD25^+^ and ST2^+^ CD25^+^ ILC2s among 7-aminoactinomycin D-negative, lineage marker-negative CD45^+^ cells were analyzed on a MACSQuant (Miltenyi Biotec) with FlowJo software (Tree Star).

### Statistics

Unless otherwise specified, ANOVA and the unpaired Student’s t test, two-tailed, were used for statistical evaluation of the results. All results are shown as means + SEM. P values of less than 0.05 were considered statistically significant using GraphPad Prism software (San Diego, CA).

## References

[1] Galli SJ, Tsai M, Piliponsky AM (2008). The development of allergic inflammation. Nature.

[2] Lambrecht BN, Hammad H (2014). Allergens and the airway epithelium response: gateway to allergic sensitization. J Allergy Clin Immunol.

[3] Sporik R, Holgate ST, Platts-Mills TA, Cogswell JJ (1990). Exposure to house-dust mite allergen (Der p I) and the development of asthma in childhood. A prospective study. N Engl J Med.

[4] Rosenstreich DL (1997). The role of cockroach allergy and exposure to cockroach allergen in causing morbidity among inner-city children with asthma. N Engl J Med.

[5] Knutsen, A. P. *et al*. Fungi and allergic lower respiratory tract diseases. *J Allergy Clin Immunol* **129**, 280–291; quiz 292–283, 10.1016/j.jaci.2011.12.970 (2012). 10.1016/j.jaci.2011.12.970 22284927

[6] Arizmendi NG (2011). Mucosal allergic sensitization to cockroach allergens is dependent on proteinase activity and proteinase-activated receptor-2 activation. J Immunol.

[7] Kheradmand F (2002). A protease-activated pathway underlying Th cell type 2 activation and allergic lung disease. J Immunol.

[8] Kouzaki H, O’Grady SM, Lawrence CB, Kita H (2009). Proteases induce production of thymic stromal lymphopoietin by airway epithelial cells through protease-activated receptor-2. J Immunol.

[9] Xiao C (2011). Defective epithelial barrier function in asthma. J Allergy Clin Immunol.

[10] Dinarello CA (2018). Overview of the IL-1 family in innate inflammation and acquired immunity. Immunol Rev.

[11] Chua KY (1988). Sequence analysis of cDNA coding for a major house dust mite allergen, Der p 1. Homology with cysteine proteases. J Exp Med.

[12] Milne J, Brand S (1975). Occupational asthma after inhalation of dust of the proteolytic enzyme, papain. Br J Ind Med.

[13] Oboki K (2010). IL-33 is a crucial amplifier of innate rather than acquired immunity. Proc Natl Acad Sci USA.

[14] Motomura Y (2014). Basophil-derived interleukin-4 controls the function of natural helper cells, a member of ILC2s, in lung inflammation. Immunity.

[15] Halim TY, Krauss RH, Sun AC, Takei F (2012). Lung natural helper cells are a critical source of Th2 cell-type cytokines in protease allergen-induced airway inflammation. Immunity.

[16] Morita H (2015). An Interleukin-33-Mast Cell-Interleukin-2 Axis Suppresses Papain-Induced Allergic Inflammation by Promoting Regulatory T Cell Numbers. Immunity.

[17] O’Connor GT (2004). Airborne fungi in the homes of children with asthma in low-income urban communities: The Inner-City Asthma Study. J Allergy Clin Immunol.

[18] Shelton BG, Kirkland KH, Flanders WD, Morris GK (2002). Profiles of airborne fungi in buildings and outdoor environments in the United States. Appl Environ Microbiol.

[19] O’Driscoll BR, Hopkinson LC, Denning DW (2005). Mold sensitization is common amongst patients with severe asthma requiring multiple hospital admissions. BMC Pulm Med.

[20] Bush RK, Portnoy JM, Saxon A, Terr AI, Wood RA (2006). The medical effects of mold exposure. J Allergy Clin Immunol.

[21] Denning DW (2014). Fungal allergy in asthma-state of the art and research needs. Clin Transl Allergy.

[22] Kiss A (2007). A new mechanism regulating the initiation of allergic airway inflammation. J Allergy Clin Immunol.

[23] Yang XO (2008). Regulation of inflammatory responses by IL-17F. J Exp Med.

[24] Nakae S (2013). Role of interleukin-33 in innate-type immune cells in allergy. Allergol Int.

[25] Kabata H, Moro K, Koyasu S, Asano K (2015). Group 2 innate lymphoid cells and asthma. Allergol Int.

[26] van Rijt L, von Richthofen H, van Ree R (2016). Type 2 innate lymphoid cells: at the cross-roads in allergic asthma. Semin Immunopathol.

[27] Kondo Y (2008). Administration of IL-33 induces airway hyperresponsiveness and goblet cell hyperplasia in the lungs in the absence of adaptive immune system. Int Immunol.

[28] Kurowska-Stolarska M (2008). IL-33 induces antigen-specific IL-5+ T cells and promotes allergic-induced airway inflammation independent of IL-4. J Immunol.

[29] Suzukawa M (2012). Epithelial cell-derived IL-25, but not Th17 cell-derived IL-17 or IL-17F, is crucial for murine asthma. J Immunol.

[30] Nakanishi W (2013). IL-33, but not IL-25, is crucial for the development of house dust mite antigen-induced allergic rhinitis. PLoS One.

[31] Ishii A (2010). Development of IL-17-mediated delayed-type hypersensitivity is not affected by down-regulation of IL-25 expression. Allergol Int.

[32] Carpino N (2004). Absence of an essential role for thymic stromal lymphopoietin receptor in murine B-cell development. Mol Cell Biol.

[33] Nakae S (2007). TNF can contribute to multiple features of ovalbumin-induced allergic inflammation of the airways in mice. J Allergy Clin Immunol.

[34] Hiraishi Y (2016). TIM-3 is not essential for development of airway inflammation induced by house dust mite antigens. Allergol Int.

[35] Morita H (2012). ST2 requires Th2-, but not Th17-, type airway inflammation in epicutaneously antigen- sensitized mice. Allergol Int.

